# Retraction: Trop2 promotes proliferation, invasion and EMT of nasopharyngeal carcinoma cells through the NF-κB pathway

**DOI:** 10.1039/d2ra90019j

**Published:** 2022-03-02

**Authors:** Nan Cheng, Haixia Li, Junpeng Luo

**Affiliations:** Department of Otolaryngological, Huaihe Hospital of Henan University Kaifeng 475000 China; Department of Oncology, Huaihe Hospital of Henan University No. 8, Baobei Road, Gulou District Kaifeng 475000 China pengluoun@163.com +86-371-23906055

## Abstract

Retraction of ‘Trop2 promotes proliferation, invasion and EMT of nasopharyngeal carcinoma cells through the NF-κB pathway’ by Nan Cheng *et al.*, *RSC Adv.*, 2017, **7**, 53087–53096, DOI: 10.1039/c7ra09915k.

The authors hereby wholly retract this *RSC Advances* article due to concerns with the reliability of the data. The authors have attempted to replicate the experiments described in the article but were unable to achieve the same results.

Given the lack of reliable raw data, the authors can no longer guarantee that the findings presented in this paper are accurate.

Signed: Junpeng Luo

Date: 24^th^ February 2022

Retraction endorsed by Laura Fisher, Executive Editor, *RSC Advances*

## Supplementary Material

